# The explanatory role of sedentary screen time and obesity in the increase of chronic back pain amongst European adolescents: The HBSC study 2002–2014

**DOI:** 10.1002/ejp.2003

**Published:** 2022-07-05

**Authors:** Josep Roman‐Juan, Rubén Roy, Mark P. Jensen, Jordi Miró

**Affiliations:** ^1^ Universitat Rovira i Virgili Unit for the Study and Treatment of Pain – ALGOS Research Center for Behavior Assessment (CRAMC) Department of Psychology Catalonia Spain; ^2^ Institut d'Investigació Sanitària Pere Virgili Universitat Rovira i Virgili Catalonia Spain; ^3^ Department of Rehabilitation Medicine University of Washington Seattle Washington USA

## Abstract

**Background:**

Previous research has shown that chronic back pain amongst European adolescents is increasing. Determining the factors associated with this increasing trend is crucial for developing prevention strategies. In this study, we used data from the Health Behaviour in School‐aged Children (HBSC) survey to examine whether increases in screen time and/or obesity between 2002 and 2014 were associated with the increase in the prevalence of chronic back pain amongst European adolescents during the 12‐year period.

**Methods:**

Data from 423,092 adolescents from 27 European countries/regions were drawn from the HBSC questionnaire‐based surveys conducted in 2002, 2006, 2010 and 2014. The Karlson–Holm–Breen method was used to examine the explanatory role of increases in screen time and obesity on the increase in the prevalence of chronic back pain whilst controlling for sex and age.

**Results:**

Increases in both screen time and obesity between 2002 and 2014 were associated with increases in the prevalence of chronic back pain (*p* < 0.001). The percent of chronic back pain prevalence increase accounted for by screen time and obesity was 3.98% and 1.65%, respectively.

**Conclusions:**

The increase in the prevalence of chronic back pain amongst European adolescents may be explained, in part, by the rising trends in both sedentary screen time and obesity. The fact that screen time and obesity only accounted for a small part of the increase in the prevalence of chronic back pain indicates that other unmeasured factors also play a role.

**Significance:**

More screen time and obesity are slightly associated with more chronic back pain (CBP) prevalence in adolescents across the WHO European Region. The findings may be used to identify ways to prevent or reduce the rising trend of CBP in adolescents.

## 1 INTRODUCTION

Research has shown that chronic back pain interferes with function in adolescents (Bejia et al., 2005; Huguet et al., 2009; Jones et al., 2004) and has an enormous societal impact (Groenewald et al., 2015; Ochsmann et al., 2010). Research has also shown that adolescents with low back pain are more likely to experience low back pain in their adulthood (Brattberg, 2004; Hestbaek, Leboeuf‐Yde, & Kyvik, 2006; Hestbaek, Leboeuf‐Yde, Kyvik, & Manniche, 2006; Jeffries et al., 2007). Moreover, low back pain causes more global disability than any other of the 291 conditions studied in the Global Burden of Disease Study (Hoy et al., 2014; Wu et al., 2020).

Recent findings show that the prevalence of chronic back pain amongst European adolescents is increasing. For example, Roy and colleagues (Roy et al., 2021) examined pooled data from 33 countries, mainly European, of the Health Behaviour amongst School‐aged Children (HBSC) cross‐sectional survey conducted from 2002 to 2014 and found that the prevalence of self‐reported chronic back pain had steadily increased from 18.3% to 21.6% across the 12‐year period. Determining the factors that account for such an increase in the prevalence of chronic back pain amongst children and adolescents is important for policymakers to improve the development and implementation of preventive and treatment strategies.

Another recent report from the World Health Organization Regional Office for Europe using data from the HBSC study (Inchley et al., 2017) revealed two additional worrisome findings. First, there has been a rapid increase (from 2002 to 2014) in the time adolescents have been spending in sedentary screen‐based activities, with almost 90% of European adolescents exceeding the recommendations of 2‐h screen time daily in 2014 (Ghekiere et al., 2018). Second, the number of obese adolescents had also increased in many European countries and regions during this period, reaching an average prevalence of 19% of adolescents above the normal weight in 2014 (Inchley et al., 2017).

Time spent in sedentary screen‐based activities—such as computer use, tablet, smartphones and TVs—has been repeatedly associated with multiple health complaints and conditions, including back pain, in adolescents (Bento et al., 2020; Hakala et al., 2012; Joergensen et al., 2021; Keane et al., 2017; Lemes et al., 2021; Silva et al., 2017; Torsheim et al., 2010). There is also mounting evidence showing a significant positive association between obesity and low back pain in adolescents (Hershkovich et al., 2013; Hestbaek, Leboeuf‐Yde, & Kyvik, 2006; Hestbaek, Leboeuf‐Yde, Kyvik, & Manniche, 2006; Palmer et al., 2020; Paulis et al., 2014). Considering these findings as a group, it is reasonable to hypothesize that the increasing rates of screen time and obesity may be associated, in part at least, with the increase of chronic back pain prevalence in European adolescents. Here, we used data from the international HBSC survey collected during the four most recent waves (2001/2, 2005/6, 2009/10 and 2013/14) to test this hypothesis.

## 2 METHOD

### 2.1 Study design, setting and sample

The Health Behaviour amongst School‐aged Children (HBSC) study is a World Health Organization collaborative cross‐sectional study that aims to monitor the health and well‐being of adolescents across Europe and North America using a standardized methodological approach (Currie et al., 2014). Data have been collected every 4 years since 1983/1984 in school settings from independent, nationally representative samples of 11‐, 13‐ and 15‐year‐old boys and girls in each participating country/region. Participants are recruited via multistage stratified random cluster sampling, with school classes or the school as the sampling unit. Each country/region obtained approval to conduct the survey from an ethics review board or a country/region‐specific equivalent regulatory body. Further information about the methodology of the HBSC study is available elsewhere (Currie et al., 2014; Roberts et al., 2009). For this study, secondary data from 801,648 adolescents were retrieved from four consecutive waves, covering the period between 2002 and 2014 (i.e. 2001/2, 2005/6, 2009/10 and 2013/14).

### 2.2 Measures

#### 2.2.1 Chronic back pain

The presence of chronic back pain was extracted from participants' responses to one of the questions of the HBSC Symptom Checklist (HBSC‐SCL), an 8‐item symptom checklist developed for the HBSC survey which has been found to provide reliable and valid measures of subjective health complaints (Haugland & Wold, 2001). The HBSC‐SCL asks the participant to respond to the question ‘In the last 6 months: how often have you had the following…?’ on a variety of physical and emotional symptoms (i.e. headache, stomach ache, backache, dizziness, feeling low, irritability/bad temper, nervousness and sleeping difficulties). Answer categories for each question are as follows: (1) ‘About every day,’ (2) ‘More than once a week,’ (3) ‘About every week,’ (4) ‘About every month,’ and (5) ‘Rarely or never.’ For the purposes of this study, adolescents who reported having back pain weekly or more often over the last 6 months were considered to have chronic back pain. This dichotomization has been previously used in studies also using items from the HBSC‐SCL (Haugland & Wold, 2001; Roy et al., 2021).

#### 2.2.2 Screen time

The screen time behaviour was extracted from the participants' responses to three different questions: (1) ‘How many hours a day, in your free time, do you usually spend watching TV, videos, DVDs and other entertainment on a screen?’; (2) ‘How many hours a day, in your free time, do you usually spend playing games on a computer, games console, tablet (like iPad) or smartphone or other electronic devices (not only including moving or fitness games)?’ and (3) ‘How many hours a day, in your free time, do you usually spend using electronic devices such as computers, tablets (like iPad) or smartphones for other purposes, for example, homework, emailing, tweeting, Facebook, chatting, surfing the internet?’ Each question asked participants about their screen behaviour separately for weekdays and weekends with nine response options: (1) ‘None at all,’ (2) ‘About half an hour,’ (3) ‘About 1 hour a day,’ (4) ‘About 2 hours a day,’ (5) ‘About 3 hours a day,’ (6) ‘About 4 hours a day,’ (7) ‘About 5 hours a day,’ (8) ‘About 6 hours a day,’ and (9) ‘About 7 or more hours a day.’ Participants’ answers to the three questions were treated as continuous (i.e. ‘None at all’ = 0, ‘Half an hour a day’ = 0.5, ‘About an hour a day’ = 1.0, etc.) in order to compute a sum score measuring screen time for week and weekend days separately. This sum score was then used to create a weighted average mean (i.e. mean = [5 × weekday +2 weekend day]/7 days; Rey‐López et al., 2010) to reflect the average daily screen time. A cutoff at 2 h/day was set to extract an additional dichotomized variable to conform the current international health guidelines for screen time, that is exceeding 2‐h daily screen time (coded as ‘1’) or not (coded as ‘0’; American Academy of Pediatrics: Children, adolescents and television, 2001).

#### 2.2.3 Obesity

The participants' self‐reported body height (cm) and body weight (kg) were used to calculate participants' body mass index (BMI) according to the kg/m^2^ formula. We then used the WHO's gender‐specific BMI‐for‐age growth charts (De Onis et al., 2007) to determine adolescents' weight status according to their chronological age (i.e. the difference between the date of administration of the HBSC questionnaire and participants' self‐reported month and year of birth). Obesity was defined by the >97th percentile on the gender‐specific BMI‐for‐age growth charts (De Onis et al., 2007; De Onis & Lobstein, 2010).

### 2.3 Data analysis

Because this study focused on European countries, we first eliminated data belonging to participants from non‐European countries (i.e. Canada and the United States of America). We also eliminated data from European countries that did not participate in the four survey waves (i.e. Albania, Armenia, Bulgaria, Iceland, Luxemburg, Malta, Moldova, Romania, Slovakia and Turkey) and from European countries that did not provide data on each variable of interest for each of the four survey waves (i.e. Austria, Czech Republic, Greenland, Norway and Ukraine). We then removed data from respondents who had not provided answers to all the study variables. For the remaining participants, we computed the number and percentages of all categorical variables, and mean and standard deviations of all continuous variables, across the four measurement periods (i.e. 2002, 2006, 2010 and 2014) for descriptive purposes. Logistic regression models including survey year as a continuous variable and sex and age as control variables were fitted to evaluate the significance of time trends for each of the three outcomes (i.e. screen time, obesity and chronic back pain). Then, to address the study's aim, for chronic back pain, a second model was run that included screen time and obesity to examine the explanatory role of both variables on the trend of chronic back pain. Additionally, the interaction term between screen time and obesity was introduced in a third model to study if the interaction contributed to the explanation of chronic back pain over time. The Karlson‐Holm‐Breen (KHB) method (Breen et al., 2013; Kohler et al., 2011) was used to decompose the total effect of survey year (i.e. time) on chronic back pain into its direct part (unmediated) and indirect part (mediated) running through screen time and obesity whilst adjusting for sex and age. This method also displays the decomposition of the indirect effect through multiple mediators and calculates the proportion of the total association that is due to each mediator (Kohler et al., 2011). The KHB method is appropriate for examining mediation in non‐linear models (Breen et al., 2013; Kohler et al., 2011) and has previously been used to examine the contribution of diverse factors (e.g. daily smoking, truancy) on the decreasing trend of heavy episodic drinking in Nordic countries (Raitasalo et al., 2021). In our study, the KHB method provided an estimate of the contribution of screen time and obesity on the increasing trend of chronic back pain. In all analyses, cluster‐robust standard errors (adjusting for adolescents being nested within countries) were used. Statistical significance was set at *p* < 0.05.

All statistical analyses were conducted using STATA 14 (StataCorp.).

## 3 RESULTS

### 3.1 Sample characteristics

As noted previously, we first removed data belonging to participants from the two non‐European countries (*n* = 54,332), from the 10 European countries that did not participate in the four waves that are the focus of this study (*n* = 115,373) and from the five European countries that did not provide data on each variable of interest for each of the four survey waves (*n* = 78,705). Data from the remaining respondents who had not provided answers to all the study variables were also removed (*n* = 130,146). Thus, the final sample consisted of 423,092 adolescents (52.77% of the total/initial sample) from 27 European countries or regions (M_*age*_ = 13.67; SD_age_ = 1.65; Range = 10.16–17.16; girls = 51.21%). As shown in Table 1, there was a similar sample size amongst the four survey waves and the age‐ and sex‐related groups. Table 2 provides an overview of the prevalence of screen time, obesity and chronic back pain across the 4 survey years.

**TABLE 1:** Sample descriptives

Age	Mean: 13.67 years (SD: 1.65 years)		
		*N*	%
Age groups	11 years old	130,257	30.79
	13 years old	143,610	33.94
	15 years old	149,225	35.27
Sex	Females	216,706	51.22
	Males	206,386	48.78
Year of survey	2002	107,791	25.48
	2006	108,842	25.73
	2010	102,687	24.27
	2014	103,772	24.53
Country or region of residence	Belgium (Flemish)	16,508	3.90
	Belgium (French)	12,390	2.93
	Croatia	19,231	4.55
	Denmark	14,882	3.52
	England	8837	2.09
	Estonia	14,702	3.47
	Finland	21,080	4.98
	France	22,588	5.34
	Germany	19,035	4.50
	Greece	15,381	3.64
	Hungry	14,485	3.42
	Ireland	4652	1.10
	Israel	14,526	3.43
	Italy	14,980	3.54
	Latvia	15,500	3.66
	Lithuania	15,335	3.62
	Netherlands	14,331	3.39
	Macedonia	14,621	3.46
	Poland	19,009	4.49
	Portugal	13,578	3.21
	Russian Federation	22,072	5.22
	Scotland	8600	2.03
	Slovenia	18,000	4.25
	Spain	21,264	5.03
	Sweden	17,921	4.24
	Switzerland	19,681	4.65
	Wales	9903	2.34

**TABLE 2:** Prevalence of screen time, obesity and chronic back pain across the four survey waves

	2002	2006	2010	2014
Mean screen hours per day (SD)	3.99 (2.40)	5.58 (3.66)	6.05 (3.87)	6.40 (4.28)
Screen time (% exceeding 2 h/day)	80.18	87.77	89.76	88.59
BMI (mean; SD)	19.13 (3.22)	19.39 (3.29)	19.50 (3.39)	19.56 (3.46)
Obesity (% PC > 97)	4.02	4.45	5.18	5.25
Back pain (%)				
Rarely or never	65.05	64.27	62.26	59.04
About every month	16.89	16.83	17.84	18.76
About every week	7.72	7.81	8.46	9.26
More than once a week	5.82	6.08	6.22	7.07
About every day	4.52	5.00	5.22	5.87
Chronic back pain (%)	18.06	18.89	19.90	22.20

Abbreviation: BMI, body mass index.

### 3.2 Regression analysis

Logistic regression analysis showed a significant albeit small linear increase over time (i.e. survey year) in screen time (*β* = 0.059; *p* < 0.001; 95%IC = 0.035–0.085) and obesity (*β* = 0.025; *p* < 0.001; 95%IC = 0.012–0.038).

Results for chronic back pain are presented in Table 3. Logistic regression analysis showed a linear increase over time in the prevalence of chronic back pain (Model 1), and screen time and obesity were associated with an increased likelihood of chronic back pain (Model 2). In addition, by adding screen time and obesity to the model, the survey year effect was slightly reduced but remained significant (Model 2). Results of the interaction effect between screen time and obesity (Model 3) did not emerge as significant; therefore, they are not reported here.

**TABLE 3:** Association between survey year, screen time, obesity and chronic back pain

	Model 1			Model 2		
	*β*	95%CI	*p*‐value	*β*	95%CI	*p*‐value
Survey year	0.021	0.015–0.027	<0.001	0.019	0.014–0.026	<0.001
Screen time (ref = less than 2 h/day)				0.125	0.075–0.175	<0.001
Obesity (ref = nonobese)				0.314	0.258–0.370	<0.001

*Note*: Findings adjusted for sex and age.

Table 4 displays the mediation of the association between time (i.e. survey year) and chronic back pain through screen time and obesity whilst controlling for sex and age. The model demonstrates a significant indirect and direct effect of survey year on chronic back pain, showing that screen time and obesity explained, in part, the increase in the prevalence of chronic back pain over time. The total effect of the survey year on chronic back pain was 1.06 times larger than the direct effect, and 5.62% of the total effect was due to screen time and obesity.

**TABLE 4:** Decomposition of the total effects of the survey year on chronic back pain

	*β* (95% CI)	*p*‐value
Survey year		
Total	0.021 (0.015–0.027)	<0.001
Direct	0.019 (0.014–0.026)	<0.001
Indirect	0.001 (0.000–0.002)	<0.001
Components of the difference		
	*β* (S.E.)	
Screen time	0.0008412 (0.000245)	
Obesity	0.0003485 (0.000092)	

*Note*: Findings adjusted for sex and age.

Table 5 displays the analysis disentangling the mediating effects of screen time and obesity on the trend in chronic back pain. The contribution of each mediator to the indirect effect is displayed in the ‘Indirect effect’ column, and the column labelled ‘Confounding percentage’ shows how much the effect of time (i.e. survey year) is due to each mediator.

**TABLE 5:** The contribution of screen time and obesity on the trend of chronic back pain

	Indirect effect (%)	Confounding percentage (%)
Screen time	70.71	3.98
Obesity	29.29	1.65

*Note*: Findings adjusted for sex and age.

## 4 DISCUSSION

The key finding of this study is that the rising trends in sedentary screen time and obesity are slightly associated with an increase in the prevalence of chronic back pain in European adolescents. The data showed that sedentary screen time accounted for up to 3.98% of the variance in the upward trend of chronic back pain, and obesity accounted for up to 1.65%. These results are consistent with those from previous studies showing positive significant associations between back pain in adolescents and both screen time (e.g. Bento et al., 2020; Keane et al., 2017; Lemes et al., 2021; Silva et al., 2017; Torsheim et al., 2010) and obesity (e.g. Hershkovich et al., 2013; Hestbaek, Leboeuf‐Yde, & Kyvik, 2006; Hestbaek, Leboeuf‐Yde, Kyvik, & Manniche, 2006; Palmer et al., 2020; Paulis et al., 2014). The results of this study confirm and extend preliminary findings with a wider, international and heterogeneous sample of adolescents.

These findings have relevant public health and economic implications. If the trend for increasing obesity and sedentary screen time in adolescents keeps growing, as reported by recent studies (e.g. Ghekiere et al., 2018; Inchley et al., 2017), then is more likely that the number of adolescents with chronic back pain will also increase. In this event, an increase in adults with chronic back pain could also be anticipated, given that adolescents with a history of back pain are more likely to have back pain in their adulthood (Brattberg, 2004; Hestbaek, Leboeuf‐Yde, & Kyvik, 2006; Hestbaek, Leboeuf‐Yde, Kyvik, & Manniche, 2006; Jeffries et al., 2007). If the findings are confirmed in future studies, this growing trend in the prevalence of chronic back pain in adolescents (and adults) will contribute to an increase of needed resources at the education, health and economic levels of the European countries and regions. Indeed, more healthcare professionals and specialized treatment programmes will be required to meet the needs of the growing number of individuals with chronic back pain. When implemented, these actions will lead to a greater demand for economic resources to address the increase in spending.

The findings of this study provide additional support for the importance of implementing public health measures to reduce sedentary screen time and prevent obesity in youth. In this regard, the promotion of a more physically active lifestyle amongst adolescents is crucial. The benefits of regular moderate‐to‐vigorous physical activity for adolescents are numerous, including a decrease in depressive symptoms, reduced blood pressure and obesity and increased bone mineral density (Janssen & LeBlanc, 2010). Despite the limited research in this field, a recent systematic review of observational studies found moderate evidence for a U‐shape relationship between physical activity and low back pain in children and adolescents (Kędra et al., 2020), suggesting the possibility that a moderate level of physical activity has a protective effect against low back pain (Kędra et al., 2020). In fact, regular physical activity, in addition to postural education and hygiene, and engagement in formal physical therapy exercises have been suggested for the treatment and prevention of low back pain in children and adolescents (Landry et al., 2015). However, the extent to which these interventions prevent back pain in younger samples needs to be more systematically studied.

This study has several limitations that must be considered when interpreting the results. First, the data available for the current analyses were limited to just a few of the many factors that are thought to contribute to the complex experience that is chronic back pain. The fact that both the rising prevalence in sedentary screen time and obesity only accounted for a small part of the increase in the prevalence of chronic back pain amongst the participants indicates that there are other unmeasured variables which are important. We now know that the rates of mental health problems and sleep difficulties amongst children and adolescents have been increasing in the last decades (Collishaw, 2015; Fleming et al., 2014; Ghekiere et al., 2018). Research has shown that both of these are risk factors of back pain in children and adolescents (Beynon et al., 2019; Kamper et al., 2016). Additional research to evaluate the role of these and other factors play in back pain is needed. Second, this study is based on cross‐sectional data, which enables us to only establish associations amongst variables but not causation. Indeed, it could be argued that the increase in time spent in sedentary screen‐based activities is a consequence of the increase in the prevalence of chronic back pain, which, in turn, may facilitate weight gain and obesity. Therefore, longitudinal studies examining the temporal sequence, which is a necessary condition to establish causal relationships, are needed to determine the direction of the effects. Third, the study only used self‐reported measures to assess screen time, body height and body weight, and chronic back pain. Self‐report measures are known to be influenced by social desirability and recall bias. Although self‐reported information about these variables is also known to have acceptable validity and reliability (Aasvee et al., 2015; Bobakova et al., 2015; Haugland & Wold, 2001; Liu et al., 2010; Rey‐López et al., 2010; Schmitz et al., 2004), research that also included objective measures of these variables would help to increase overall trust in the findings.

Despite the study's limitation, this is the first study to our knowledge that examines the explanatory role of two specific factors in the rising trend of chronic back pain, using a representative sample of European adolescents. In addition, the finding that both increases in sedentary screen time and obesity are slightly associated with the rising rates of chronic back pain in this population provides important new information that may be useful for policymakers, healthcare professionals and researchers, as we work to evaluate and improve public health measures that can reduce the prevalence and impact of low back pain in adolescents.

## AUTHOR CONTRIBUTIONS

JR‐J originated the idea, analysed the data, interpreted the results and wrote the first draft of the manuscript. RR contributed to the statistical analysis and revised the drafting manuscript. MJ and JM contributed to the conception and design of the study and revised and edited subsequent drafts of the manuscript. All authors discussed the results and commented on the manuscript.

## FUNDING INFORMATION

This work was partly funded by grants from the Spanish Ministry of Economy, Industry and Competitiveness (RTI2018‐09870‐B‐I00; RED2018‐102546‐T), the European Regional Development Fund (ERDF), the Government of Catalonia (AGAUR; 2017SGR‐1321), Fundación Grünenthal, Universitat Rovira i Virgili (PFR program) and ICREA‐Acadèmia. JR‐J and RR are supported by doctoral grants from MINECO.

## CONFLICT OF INTEREST

The authors declare no financial or other relationships that might lead to a conflict of interest related to this study.
